# High expression of CDCA7 predicts poor prognosis for clear cell renal cell carcinoma and explores its associations with immunity

**DOI:** 10.1186/s12935-021-01834-x

**Published:** 2021-03-01

**Authors:** Shouyong Liu, Yi Wang, Chenkui Miao, Qianwei Xing, Zengjun Wang

**Affiliations:** 1 Department of Urology, The First Affiliated Hospital of Nanjing Medical University, No. 300, Guangzhou Road, Nanjing, 210029 Jiangsu Province China; 2 Department of Urology, Affiliated Hospital of Nantong University, No. 20 West Temple Road, Nantong, 226001 Jiangsu Province China

**Keywords:** CDCA7, Clear cell renal cell carcinoma, Prognosis, Survival, Immunity

## Abstract

**Background:**

Cell division cycle-associated 7 (CDCA7), as a member of the cell division cycle associated family, was reported to be aberrantly expressed in both solid tumors and hematological tumors, suggesting its essential role in promoting tumorigenesis. Hence, we aimed to explore its comprehensive roles of overall survival (OS) in clear cell renal cell carcinoma (ccRCC) and emphasize its associations with immunity.

**Methods:**

The RNA sequencing data and corresponding clinical information were downloaded from The Cancer Genome Atlas (TCGA) database. Gene set enrichment analysis (GSEA) was adopted to explore CDCA7 associated signaling pathways. Univariate and multivariate Cox regression analyses were carried out to assess independent prognostic factors. Furthermore, roles of CDCA7 in human immunity were also investigated.

**Results:**

Our results suggested that CDCA7 was overexpressed in ccRCC and its elevated expression was related to shorter OS (P < 0.01). Univariate and multivariate Cox regression analyses identified CDCA7 as an independent prognostic factor (both P < 0.05). The prognostic nomogram integrating CDCA7 expression level and clinicopathologic variables was constructed to predict 1-, 3- and 5-year OS. GSEA indicated that high CDCA7 expression was related to the apoptosis pathway, cell cycle pathway, JAK-STAT pathway, NOD like receptor pathway, P53 pathway, T cell receptor pathway and toll like receptor pathway, etc. Moreover, CDCA7 was significantly related to microsatellite instability (MSI, P < 0.001) and tumor mutational burden (TMB, P < 0.001). As for immunity, CDCA7 was remarkably associated with immune infiltration, tumor microenvironment, immune checkpoint molecules and immune pathways.

**Conclusions:**

CDCA7 could serve as an independent prognostic factor for ccRCC and it was closely related to MSI, TMB, and immunity.

## Background

According to Cancer Statistics 2020 in the United States, renal cell carcinoma (RCC) is the 6th and 8th most common cancer in males and females, respectively [1]. It is estimated that there will be 73,750 newly diagnosed cases with the male-to-female ratio approximately being 1.6:1.0, and 14,830 newly estimated deaths in 2020 [1]. Clear cell RCC (ccRCC) is the most common histological subtype, accounts for 75% of all kidney tumors [2, 3]. From 2009 to 2015, the 5-year relative survival rate for RCC in the United States is 74% [2]. In general, RCC has been increasingly recognized as a serious, worldwide public health concern.

Cell division cycle-associated 7 (CDCA7) gene, also called JPO1, located on chromosome 2q31, is a member of the cell division cycle associated family of genes and encodes a 47 kDa nuclear protein consisting of 371 amino acids [4, 5]. JPO1 was first discovered and named by Dang et al. as a differentially expressed gene in fibroblasts transfected with c-Myc gene in a representational difference analysis (RDA) carried out to explore novel putative c-Myc target genes [5, 6]. Later the Human Genome Nomenclature Committee officially named the gene JPO1 as CDCA7 according to its periodic expression in the cell cycle, reaching its peak at the G1 to S phase transition [7]. In human normal tissues, high expression levels of CDCA7 were found in small intestine, thymus, and colon whereas relatively low in bone marrow, lymph node, spleen, and peripheral leukocytes [4]. It was reported that CDCA7 was aberrantly expressed in both solid tumors and hematological tumors, including lung cancer, stomach cancer, breast cancer, colorectal cancer, lymphoma, acute myelogenous leukemia and so on [5, 8–13], suggesting its essential role in promoting tumorigenesis. CDCA7 was identified to behave as a direct c-Myc target gene [4], containing g a leucine zipper motif and a cysteine rich region that suggested it could function as a DNA binding protein [5, 9]. Besides, CDCA7 was also reported to be a direct transcriptional target of transcription factor E2F1 [11]. Intriguingly, Ye et al. demonstrated that CDCA7 could increase the expression of EZH2, an important regulator in triple-negative breast cancer (TNBC), by binding the promoter of EZH2 to enhance its transcriptional activity [9]. All of these indicated the complex functional mechanisms of CDCA7 and more efforts were required to elucidate its role in tumorigenesis.

So far, however, there has been no detailed investigation of the role of CDCA7 in RCC. In the present study, we found CDCA7 was upregulated in ccRCC and significantly associated with some clinicopathologic parameters of patients via comprehensive and systematic bioinformatic analysis of RNA sequence data downloaded from The Cancer Genome Atlas (TCGA) database. CDCA7 also could serve as an independent prognostic factor of ccRCC patients. Gene Set Enrichment Analysis (GSEA) and Kyoto Encyclopedia of Genes and Genomes (KEGG) were carried out to explore related enrichment genes and signaling pathways. Furthermore, we performed immune-related analysis of CDCA7 in ccRCC to reveal its potential function.

## Materials and methods

### Data acquisition

The RNA sequence data and related clinical information of kidney renal clear cell carcinoma (KIRC) patients were searched from The Cancer Genome Atlas (TCGA) Data Portal (http://cancergenome.nih.gov/), containing 531 ccRCC tissues and 72 matched adjacent normal kidney tissues. International Cancer Genome Consortium (ICGC) dataset (N = 45; T = 91; http://dcc.icgc.org) and ArrayExpress dataset (E-MTAB-1980; T = 99; https://www.ebi.ac.uk/arrayexpress/) were utilized as the external validation cohorts. Data were standardized by log2 transformation and we set |log2 fold change (FC)|≥ 1 and false discovery rate (FDR) < 0.05 as the cut-off value.

### RNA extraction, reverse transcription and quantitative real-time PCR (qRT-PCR)

16 pairs of ccRCC and corresponding adjacent normal kidney tissues were obtained from patients with primary ccRCC who had underwent radical nephrectomy at the Department of Urology of the First Affiliated Hospital of Nanjing Medical University. The clinical information of the 16 ccRCC patients was shown in Additional file 1: Table S1. Total RNA was extracted from clinical samples using TRIzol reagent (Invitrogen, Carlsbad, CA, USA) and cDNA was synthesized using HiScript III RT SuperMix for qPCR (+ gDNA wiper) (Vazyme, Nanjing, China) according to the manufacturer’s instructions. The qRT-PCR was performed by using StepOne Plus Real-time PCR system (Applied Biosystems, Foster City, CA, USA) with ChamQ SYBR qPCR Master Mix (High ROX Premixed) (Vazyme). The following primers were used for qRT-PCR: CDCA7, forward: 5′-GGGTGGCGATGAAGTTTCCA-3′, reverse: 5′-GGGGATGTCTTCCACGGAAC-3′; β-actin, forward: 5′-CTCGCCTTTGCCGATCC-3′, reverse: 5′-TTCTCCATGTCGTCCCAGTT-3′. Data analysis was performed with ABI Step One Software version 2.1 and the relative mRNA level was calculated using 2^−ΔΔCt^ methods.

### The Kaplan Meier survival analysis and the receiver operating characteristic analysis

The median expression value of CDCA7 in the enrolled 531 ccRCC patients was set as the cut-off value and the patients were divided into a high-risk group and a low-risk group. The Kaplan Meier survival curve was plotted to analyze the different survival outcomes of the two groups. The receiver operating characteristic curves (ROC) were generated by means of the “survivalROC” package, and the area under the curve (AUC) values were calculated to assess the specificity and sensitivity of CDCA7 and associated clinicopathologic parameters including age, gender, race, grade, stage, and T, N, M.

### Univariate and multivariate Cox hazard regression analysis

In order to identify significantly OS-related independent factors, univariate and multivariate Cox regression analyses were conducted to exclude clinical characteristics with little prognostic values from age, gender, ethnicity, grade, stage, T, N, M and CDCA7 expression level by R package.

### Construction of nomogram model

In order to predict the possibility of OS and visualize the correlation between individual predictors (age, gender, ethnicity, grade, stage, T, M, N) and survival outcomes, we applied a nomogram model to help clinicians observe and predict the prognosis of ccRCC patients by using the R “rms” package. Points were divided to every parameter by performing the point scale in the nomogram, and we calculated the whole points by summing up the points of all factors.

### Gene set enrichment analysis (GSEA)

To analyze the signaling pathways of CDCA7, Kyoto Encyclopedia of Genes and Genomes (KEGG) pathway enrichment analysis was performed with the help of “clusterProfiler” R package. Gene Set Enrichment Analysis (GSEA) is a powerful analytical method used to interpret gene expression data and analyze statistically significant and consistent differences between different groups with different biological states [14]. The permutation tests were carried out 1000 times for discovering significant critical biological pathway. It had been considered to be significantly enriched that nominal p value less than 0.05 and FDR < 25%.

### The evaluation of CDCA7 in microsatellite instability (MSI), tumor mutational burden (TMB) and neoantigen

With the help of MISA (http://pgrc.ipk-gatersleben.de/misa/misa.html), we screened out all the autosomal microsatellite tracts containing five or more repeating subunits of 1–5 bp in length as described previously [15]. On the basis of the number of somatic nonsynonymous mutations (NSM), we carried out the mutation burden analysis via the comparison of sequence data between tumor tissues and its blood samples as previously described [16]. In addition, seq2HLA version 2.2 was applied to obtain 4-digit typing data for different cancers with no change of default settings in the TCGA database. Then we carried out pvac-seq to generate specific neoantigen on the samples, especially in ccRCC [17].

### Correlation analysis of CDCA7 in immune infiltration and tumor microenvironment

To evaluate the performance of CDCA7 gene in immune infiltration, we performed the correlation analysis between CDCA7 and six immune cell infiltration with the help of the purity-adjusted Spearman. Moreover, we performed the ESTIMATE algorithm to assess tumor microenvironment-related scores including the estimate score, stromal score and immune score in ccRCC patients via normalized expression matrix [18]. P-values < 0.05 were considered to be statistically significant.

CIBERSORT is a significant deconvolution algorithm used to predict the fractions of multiple cell types by analyzing gene expression profiles of admixtures [19]. We could estimate the cellular composition of the whole tissues on the basis of standardized gene expression data from TCGA, indicating the abundant specific cell types. We evaluated the expression level of each gene to figure out the composition of genes in each cell from three aspects including immune checkpoint molecules, immune pathways and mismatch repair protein, respectively.

### Statistical analysis

We analyzed all of the statistical data and the figures by mean of SPSS 24.0 (IBM, Chicago, USA), R3.3.1 (https://www.r-project.org/) and GraphPad Prism 6.0 (San Diego, CA, USA). Pearson’s correlation method was used to analyze the correlation between two different genes. We estimated the survival predictive performance of CDCA7 with the help of Kaplan–Meier curve and log-rank test. And univariate and multivariate Cox regression analyses were utilized to assess the correlation between different variables and OS. The nomogram was generated by using the rms package of R software. ROC and AUC were performed to evaluate the prognostic ability of RS by means of the package of “survivalROC” in R. It was considered to be statistically significant that nominal P value < 0.05.

## Results

### Expression levels of CDCA7 in ccRCC

**Fig. 1:**
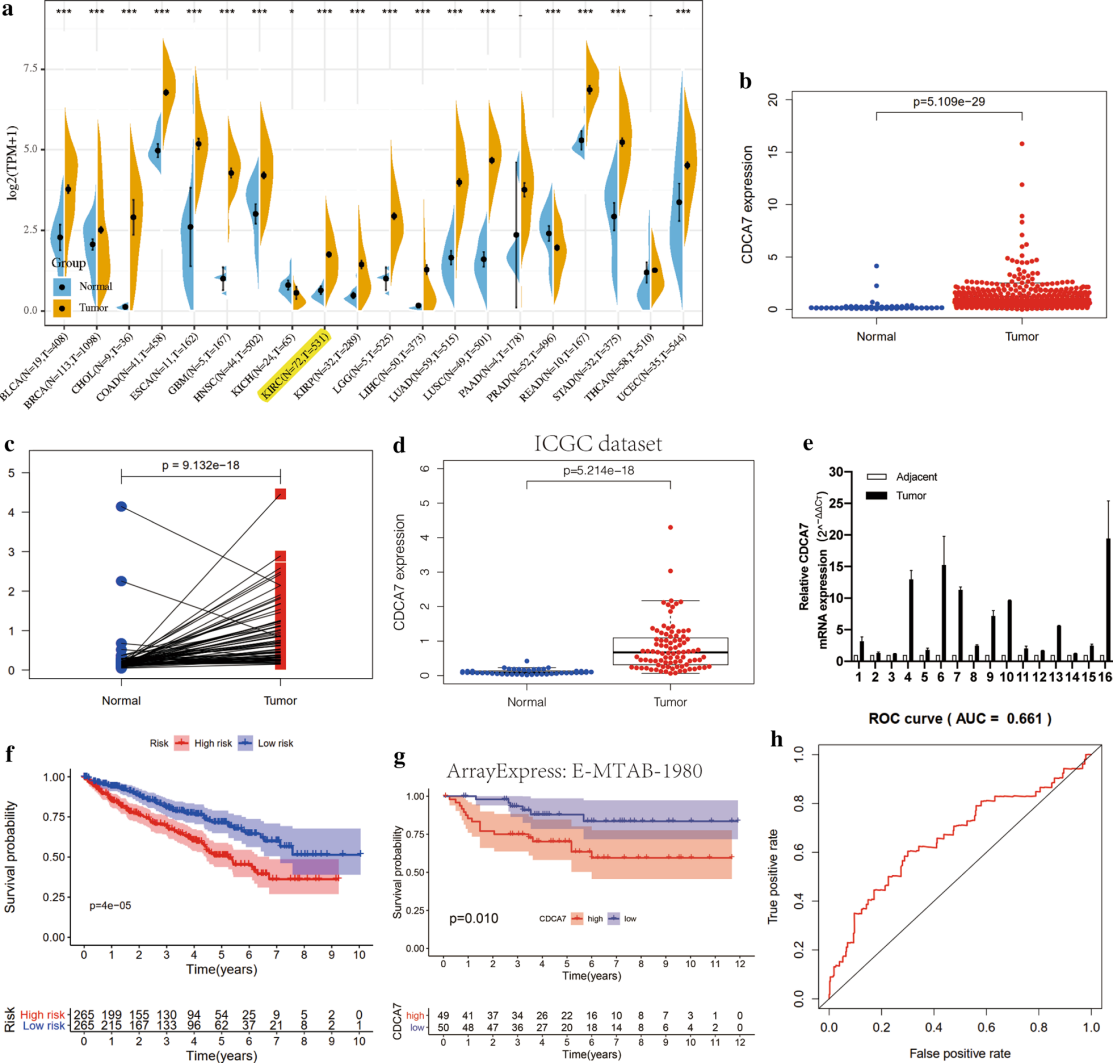
CDCA7 was overexpressed in ccRCC and associated with overall survival (OS). **a** The expression of CDCA7 in various cancers in TCGA dataset. **b** Relative expression levels of CDCA7 in ccRCC and normal kidney tissues in TCGA dataset (N = 72; T = 531). **c** Pairwise boxplot of CDCA7 expression between ccRCC and matched normal tissues in TCGA dataset (N = 72; T = 72). **d** External validation of relative expression levels of CDCA7 in ccRCC and normal kidney tissues in ICGC dataset (N = 45; T = 91). **e** CDCA7 mRNA expression in 16 pairs of ccRCC tissues and matched adjacent non-cancerous tissues. **f** Kaplan–Meier curve for low-risk and high-risk groups in TCGA database. **g** Kaplan–Meier curve for low-risk and high-risk groups in ArrayExpress database (E-MTAB-1980; T = 99). **h** ROC curve of CDCA7

We analyzed the mRNA expression levels of CDCA7 in 531 ccRCC tissues and 72 normal kidney tissues from the TCGA dataset and found that CDCA7 was upregulated in ccRCC tissues compared to the normal tissues (P < 0.001, Fig. 1a, b). The pairwise boxplot of 72 pairs of ccRCC tissues and matched adjacent normal tissues from TCGA showed most of the cancer tissues exhibited a higher level of CDCA7 (P < 0.001, Fig. 1c). We further verified the high expression level of CDCA7 in the International Cancer Genome Consortium (ICGC) dataset as external verification (P < 0.001, Fig. 1d). Furthermore, qRT-PCR results from 16 pairs of ccRCC and adjacent normal kidney tissues also exhibited a higher expression of CDCA7 in ccRCC tissues (Fig. 1e). According to the CDCA7 expression levels of the 531 ccRCC patients, we set the median expression level as the cut-off value and divided these patients into a high- and low-risk group, respectively. The Kaplan–Meier curve was plotted and showed that patients in the high-risk group had significantly poorer overall survival than those in the low-risk group (P < 0.001, Fig. 1f), suggesting its potential to predict ccRCC patients’ prognosis. Moreover, we plotted the Kaplan–Meier curve of CDCA7 in ccRCC patients with the help of ArrayExpress database (E-MTAB-1980) as external verification, which exhibited the same results (P < 0.05, Fig. 1g). The ROC analysis was performed and its AUC for CDCA7 was 0.661 (Fig. 1h), indicating the barely satisfactory prognosis predicted ability of CDCA7.

### Association of CDCA7 expression with clinicopathologic parameters

**Fig. 2:**
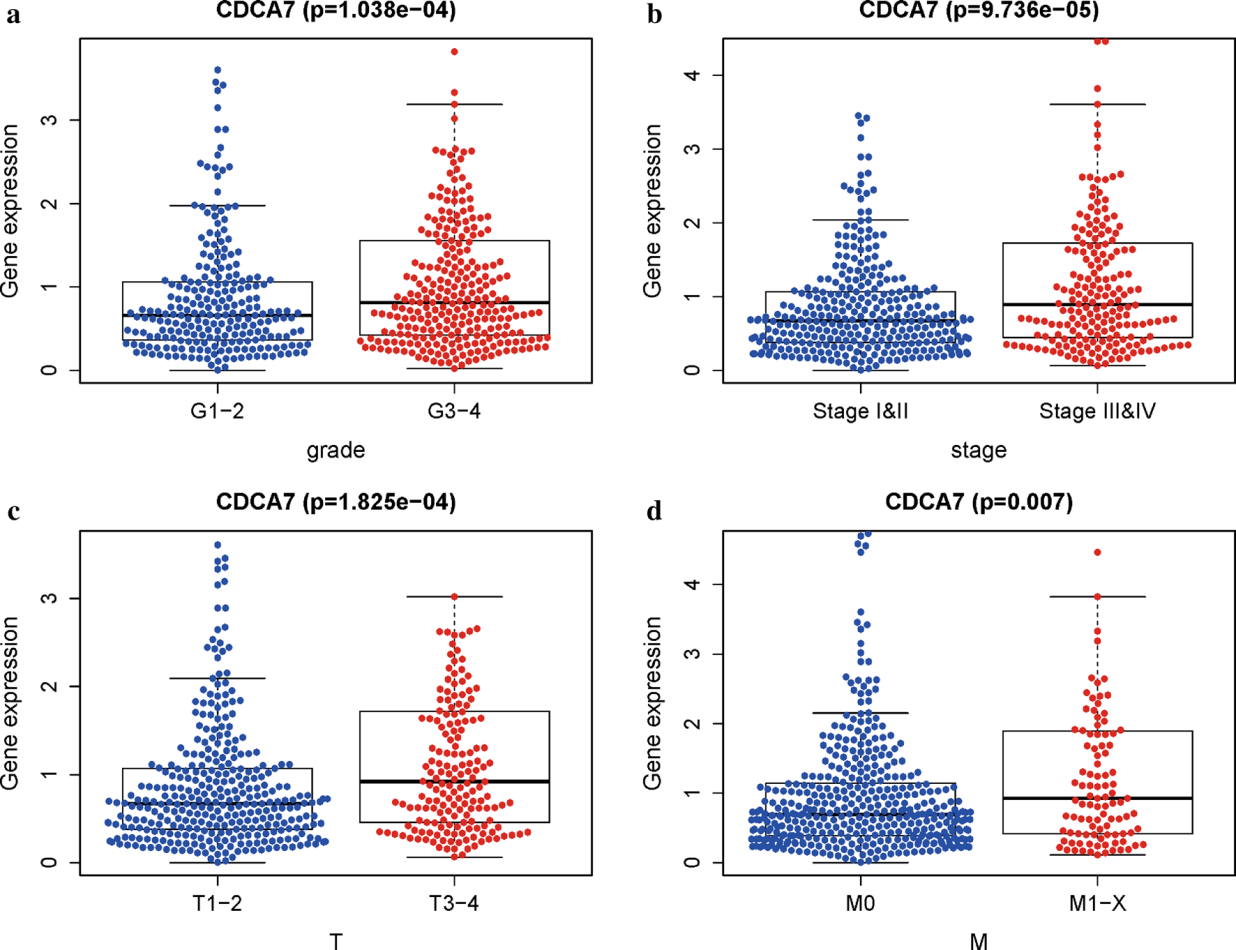
Association of CDCA7 expression with clinicopathologic characteristics. **a** Grade. **b** Stage. **c** T stage. **d** M stage

To investigate the association between CDCA7 expression and related clinicopathologic parameters, we analyzed the CDCA7 expression levels in different groups of clinicopathologic characteristics by means of independent sample t-tests. The results showed that the CDCA7 exhibited higher expression levels in groups of higher grade (P < 0.001; Fig. 2a), pathologic stage (P < 0.001; Fig. 2b), T stage (P < 0.001; Fig. 2c), and M stage (P < 0.01; Fig. 2d). No significantly difference was observed of CDCA7 expression levels in age, gender, ethnicity, and N stage (date not shown).

### Univariate and multivariate analysis of OS and construction of ccRCC prognostic prediction nomogram

**Fig. 3:**
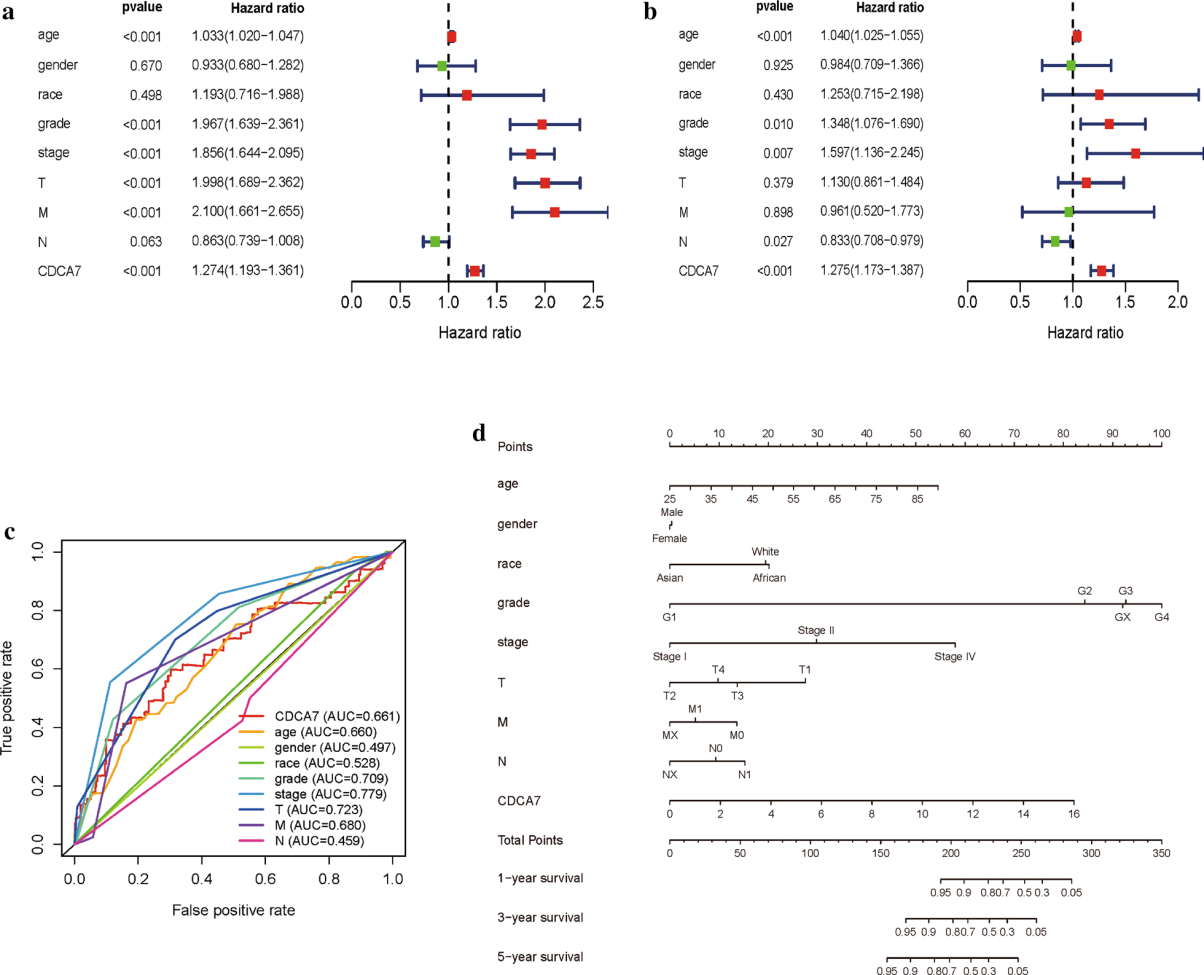
CDCA7 could serve as an independent prognostic factor and a prognostic nomogram was constructed. **a**, **b** Univariate and multivariate Cox regression analysis of clinicopathologic variables and CDCA7 in ccRCC. **c** ROC curves of CDCA7 and other clinicopathologic parameters. **d** The prognostic nomogram constructed to predict 1-, 3-, 5-year survival of ccRCC patients. *ROC* the receiver operating characteristic curves

We carried out univariate Cox and multivariate Cox regression analysis to investigate whether the CDCA7 expression was an independent prognostic factor correlated with the overall survival (OS) of ccRCC patients (Table 1). As showed in Fig. 3a, in the univariate Cox regression analysis, CDCA7 expression, grade, age, pathological stage, T stage and M stage were all significantly associated with OS of ccRCC patients. However, in the multivariate Cox regression analysis, only CDCA7 expression, grade, pathological stage, and N stage showed significant correlation with OS of ccRCC patients (Fig. 3b), and high CDCA7 expression predicted a poorer OS (HR = 1.125; P < 0.001). Based on the results above, CDCA7 could act as an independent prognostic factor of OS when adjusted by other related variables.

**Table 1:** Univariate and multivariate Cox regression analysis of CDCA7 expression level and clinicopathologic variables in ccRCC

Variable	Univariate analysis				Multivariate analysisc			
	HR	HR.95L	HR.95H	P value	HR	HR.95L	HR.95H	P value
Age (year)	1.0333	1.0197	1.0471	*0.0000*	1.0399	1.0249	1.0551	*0.0000*
Gender	0.9333	0.6797	1.2815	0.6696	0.9843	0.7090	1.3665	0.9245
Ethnicity	1.1931	0.7160	1.9881	0.4981	1.2535	0.7149	2.1977	0.4303
Grade	1.9669	1.6388	2.3606	*0.0000*	1.3483	1.0755	1.6903	*0.0096*
Stage	1.8556	1.6436	2.0950	*0.0000*	1.5974	1.1365	2.2454	*0.0070*
T	1.9976	1.6891	2.3625	*0.0000*	1.1300	0.8606	1.4836	0.3791
M	2.0996	1.6607	2.6546	*0.0000*	0.9606	0.5203	1.7735	0.8978
N	0.8630	0.7389	1.0079	0.0628	0.8328	0.7084	0.9790	*0.0267*
CDCA7	1.2742	1.1930	1.3609	*0.0000*	1.2754	1.1727	1.3871	*0.0000*

Then we carried out ROC curve analysis to assess the predictive ability of CDCA7 and other clinicopathologic parameters (Fig. 3c). The AUC of the CDCA7 expression was 0.660, higher than that of age, gender, race and lymph nodes status, and lower than that of tumor grade, pathological stage, T stage and M stage. According to the AUC results, we realized that CDCA7 expression level alone could not sufficiently predict prognosis of ccRCC patients. We further established a prognostic nomogram by integrating CDCA7 and clinicopathologic parameters (Fig. 3d). The nomogram could help to evaluate 1-, 3-, and 5-year survival probabilities to predict ccRCC patients’ prognosis with a quantitative approach.

### GSEA analysis of CDCA7

**Fig. 4:**
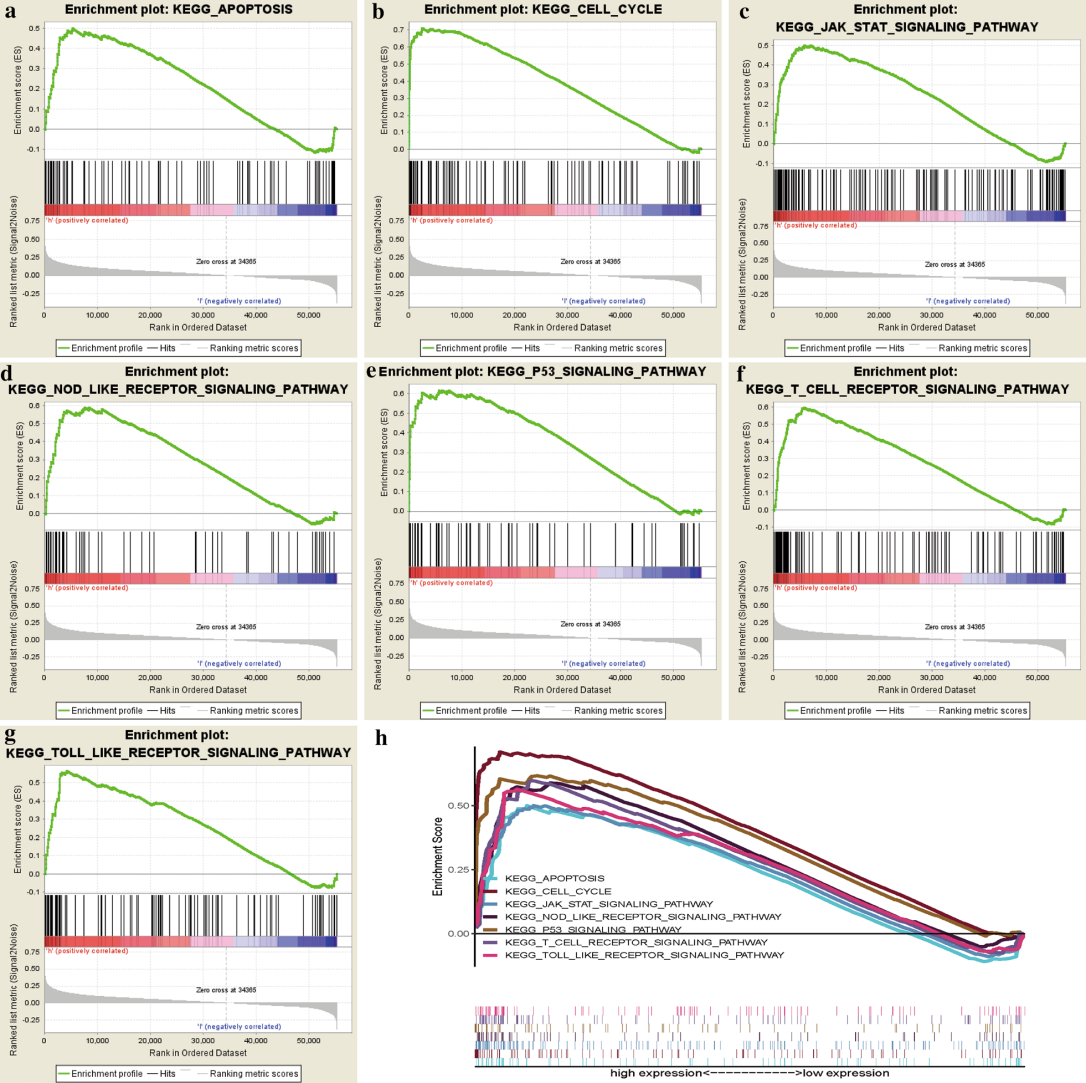
Enrichment plots from gene set enrichment analysis (GSEA). **a** Apoptosis pathway. **b** Cell cycle pathway. **c** JAK-STAT pathway. **d** NOD like receptor pathway. **e** P53 pathway. **f** T cell receptor pathway. **g** Toll like receptor pathway. **h** The seven most significantly enriched signaling pathways based on their normalized enrichment score and the expression map

Though we discovered CDCA7 could act as an independent prognostic factor in ccRCC, how CDCA7 is involved in the ccRCC pathogenesis still remained unclear. We performed GSEA analysis to explore possible mechanisms and signaling pathways through which CDCA7 functioned to regulate ccRCC. On the basis of the normalized enrichment score (NES) and FDR q-val (FDR < 0.01), the most significantly enriched biological pathways were exhibited (Table 2; Fig. 4), which were apoptosis pathway, cell cycle pathway, JAK-STAT pathway, NOD like receptor pathway, P53 pathway, T cell receptor pathway and toll like receptor pathway (Fig. 4a–h), to uncover the potential regulatory mechanism of CDCA7 in ccRCC.

**Table 2:** Gene set enrichment analysis (GSEA) of CDCA7 in ccRCC

GeneSet name	NES	Nominal P-value	FDR q-value
KEGG_apoptosis	1.8310	0.0231	0.0556
KEGG_cell_cycle	2.4277	0.0000	0.0008
KEGG_jak_stat_signaling_pathway	2.0902	0.0000	0.0115
KEGG_nod_like_receptor_signaling_pathway	2.0426	0.0038	0.0153
KEGG_P53_signaling_pathway	2.2361	0.0000	0.0043
KEGG_T_cell_receptor_signaling_pathway	2.1507	0.0039	0.0090
KEGG_toll_like_receptor_signaling_pathway	2.0946	0.0038	0.0132

### PPI network construction and association of CDCA7 with MSI, TMB, Neoantigen in ccRCC

**Fig. 5:**
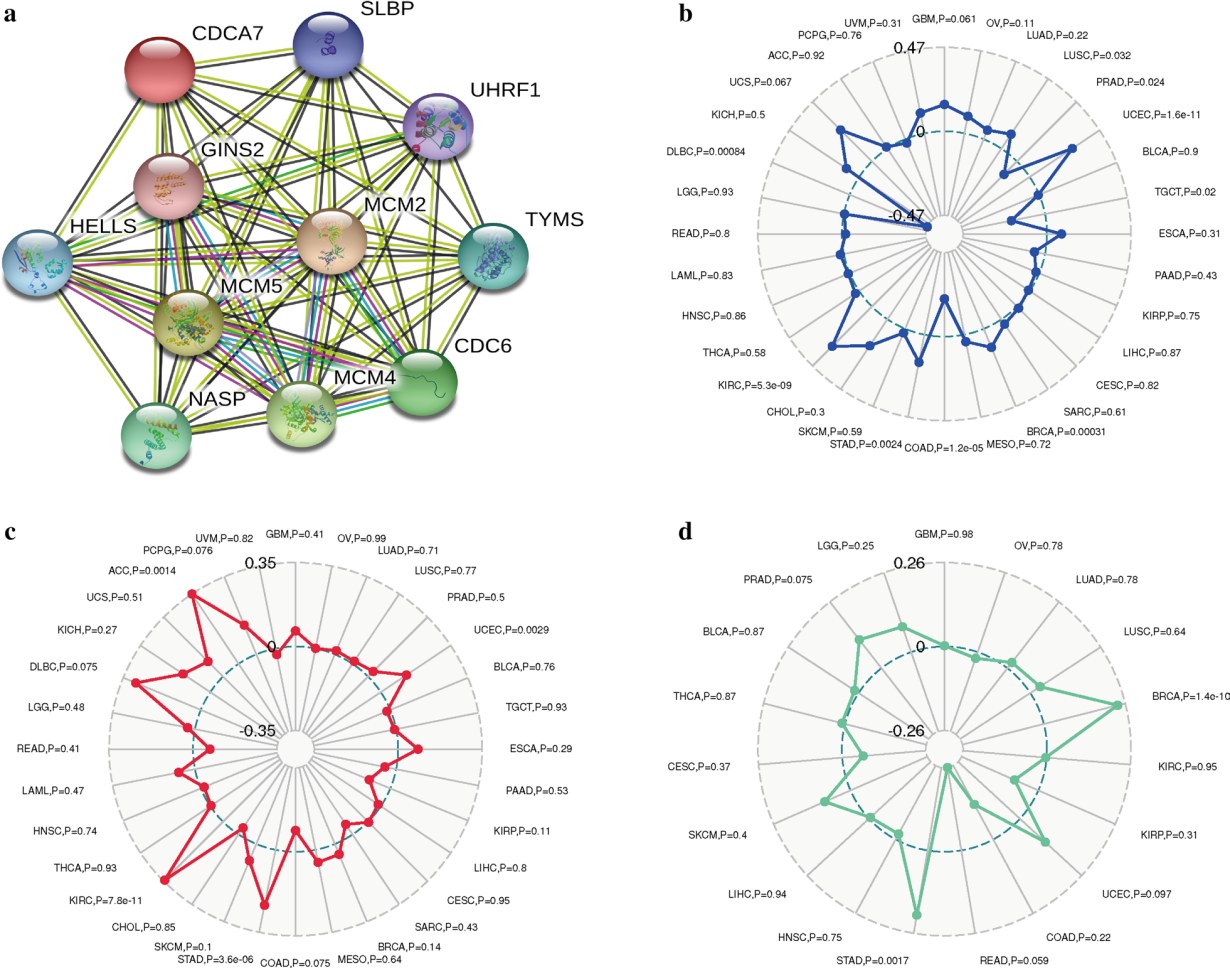
Protein–protein interaction network of CDCA7 (**a**) and associations of CDCA7 with MSI (**b**), TMB (**c**), and neoantigen (**d**). *MSI* microsatellite instability, *TMB* tumor mutational burden

To explore the potential functional interaction of CDCA7, we constructed the PPI network by applying the STRING database and the Cytoscape software. As showed in Fig. 5a, ten genes including SLBP, GINS2, HELLS, UHRF1, MCM2, MCM4, MCM5, NASP, TYMS, and CDC6 were significantly associated with CDCA7 functionally. Based on the ccRCC samples from TCGA database, we further investigated whether CDCA7 was relevant to MSI, TMB or neoantigen. The results suggested that CDCA7 was significantly related to MSI (P < 0.001, Fig. 5b) and TMB (P < 0.001, Fig. 5c), while it was not associated with neoantigen (P = 0.95, Fig. 5d).

### Associations of CDCA7 with immune infiltrations, tumor microenvironment and methyltransferase in ccRCC

**Fig. 6:**
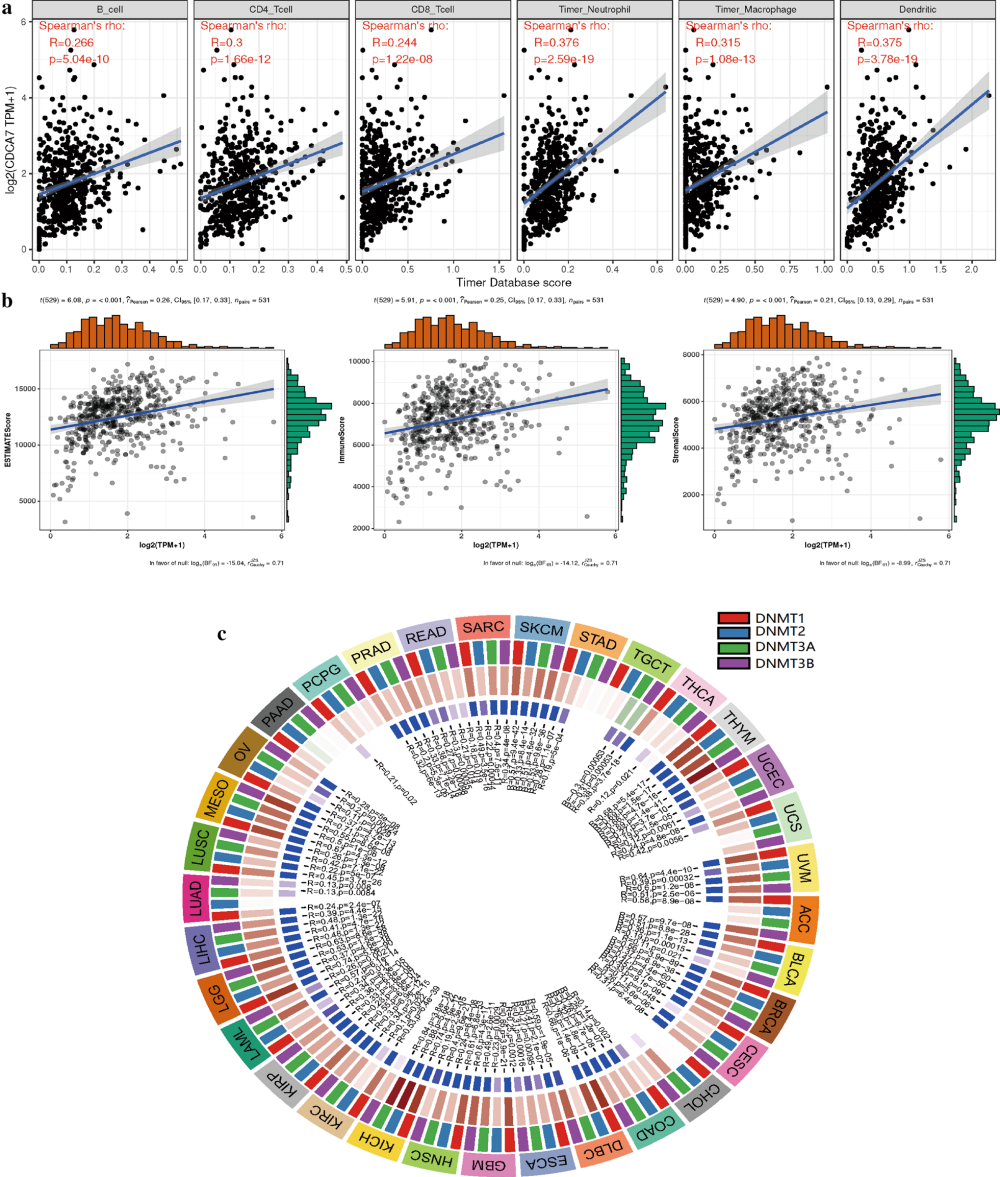
Associations of CDCA7 with immune infiltrations (**a**), immune microenvironment including immune cells, stromal cells and both of them (**b**), and methyltransferase (**c**)

Through analyzing the correlation of CDCA7 and six immune cell infiltration levels in ccRCC via online analysis TIMER, we found that in ccRCC, CDCA7 was in close connection with the immune infiltration including B cell infiltration, CD4^+^ T cell infiltration, CD8^+^ T cell infiltration, neutrophil infiltration, macrophage infiltration, and dendritic cell infiltration (P < 0.001, Fig. 6a). As to the tumor microenvironment, CDCA7 was shown to be involved in immune cells, stromal cells and both of them (P < 0.001, Fig. 6b). In addition, the DNA methyltransferase DNMT1 (P < 0.001), DNMT2 (P < 0.05), DNMT3 (P < 0.001), DNMT4 (P < 0.001) were also significantly associated with the CDCA7 expression level in ccRCC (Fig. 6c).

### Associations of CDCA7 with immune checkpoint molecules, immune pathways and mismatch repair proteins

**Fig. 7:**
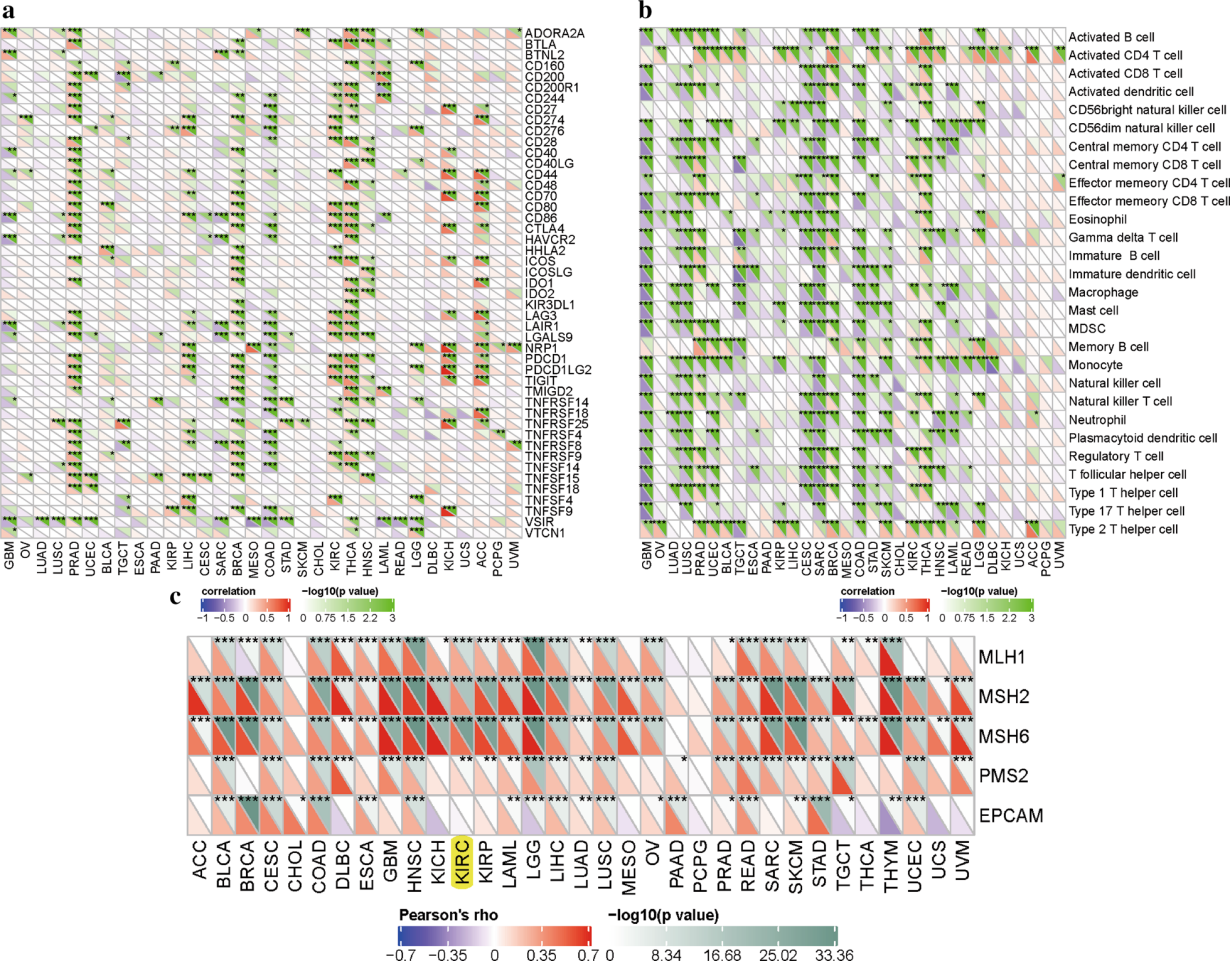
Associations of CDCA7 with immune checkpoint molecules (**a**), immune cells (**b**), and mismatch repair proteins (**c**)

In this study, we analyzed the associations of CDCA7 and 47 immune checkpoint molecules in ccRCC and finally found 25 significantly related molecules including CTLA4, CD274, LAG3 and so on (Fig. 7a). We further explored the expression levels of these 25 genes between normal kidney tissues and ccRCC tissues by utilizing the TCGA dataset and eventually identified 10 genes including LAG3, CD27, CD44, CD86, CD276, HHLA2, LAIR1, LGALS9, TIGIT, TNFRSF14 (Additional file 2: Figure S1). Besides, relationships between CDCA7 and immune pathways displayed that CDCA7 was closely linked to associated immune cells like activated CD4 T cell, regulatory T cell, memory B cell, macrophage, monocyte and so on (Fig. 7b). We also found that CDCA7 was markedly related to mismatch repair proteins including MLH1, MSH2, MSH6, PMS2 in ccRCC (Fig. 7c).

## Discussion

CDCA7 is a member of the cell division cycle associated family of genes, located on chromosome 2q31, encodes a 47 kDa nuclear protein consisting of 371 amino acids [4, 5] and is involved in embryonic development [20]. It was found that CDCA7 was expressed in many human tissues including small intestine, thymus, colon, bone marrow, lymph node, spleen, and peripheral leukocytes [4]. Previous researches had reported its close correlations to malignant tumors. As reported, CDCA7 was markedly upregulated in TNBC, the most aggressive subtype of breast cancer, related to tumor proliferation and metastatic relapse status, and predicted poor prognosis [9]. Wang etc. discovered that CDCA7 was preferentially elevated in lung adenocarcinoma (LUAD) and overexpression of CDCA7 could enhance cell proliferation in LUAD through G1 phase promotion [8]. And CDCA7 was essential for invasion and migration of lymphoma cells [10]. However, up to date, no data had been reported about the expression level and potential function of CDCA7 in ccRCC.

In our study, we carried out a systematic analysis on CDCA7 in ccRCC by analyzing the RNA sequence data downloaded from TCGA database. Comparing to the expression level of CDCA7 in normal kidney tissues, we found CDCA7 was elevated in ccRCC tissues. The enrolled ccRCC patients from TCGA were divided into a high- and low-expression group according to the median CDCA7 expression value of all the patients. And we discovered that patients in the low-expression group had longer OS than those in high. Meantime, higher expression of CDCA7 was significantly associated with higher disease grade, stage, T and M period. Then we conducted univariate and multivariate Cox regression analysis on CDCA7 and found that CDCA7 could serve as an independent prognostic factor of ccRCC. Moreover, to help clinicians predict the prognosis of ccRCC patients, we constructed a predictive nomogram based on the CDCA7 expression level and relative clinicopathological parameters.

Gene Set Enrichment Analysis (GSEA) is a computational method utilized to evaluate whether a prior defined set of genes display statistically significant, consistent differences between two biological states [21]. To explore possible signaling pathways and mechanisms CDCA7 could function through, we conducted GSEA analysis and discovered signal pathways CDCA7 might be involved in including apoptosis pathway, cell cycle pathway, JAK-STAT pathway, NOD like receptor pathway, P53 pathway, T cell receptor pathway and toll like receptor pathway. Previous researches indicated that down-expression of A100A4 could reduce cell growth of RCC via NF-kB-dependent MMP-2 and bcl-2 Pathway [22]. Fang etc. discovered that simvastatin could lead to the inhibition of cell growth of RCC via AKT/mTOR, ERK and JAK2/STAT3 pathway [23]. It was also reported that Tropomyosin-1, a widely expressed actin-binding protein, could promote cancer cell apoptosis via the p53-mediated mitochondrial pathway in ccRCC [24], and the MDM2 inhibitor MI-319 could induce RCC cell apoptosis mainly dependent on p53 overexpression [25].

Then we constructed the PPI network of CDCA7 to explore potential protein–protein interaction. It showed that ten genes were significantly functional associated with CDCA7, including SLBP, GINS2, HELLS, UHRF1, MCM2, MCM4, MCM5, NASP, TYMS, and CDC6. Researchers have discovered the important roles of these genes in the physiological process and tumor progression. For instance, SLBP (Stem-loop-binding protein) is evolutionarily conserved and involved in the processing, translation, and degradation of canonical histones mRNAs including H1, H2A, H2B, H3, and H4 [26]. With regard to GINS2, it was reported that GINS2 could promote cancer cell proliferation, migration and invasion of non-small-cell lung cancer (NSCLC) via facilitating epithelial-to-mesenchymal transition and modulating PI3K/Akt and MEK/ERK signal pathway [27]. Previous reports revealed that mutations in CDCA7 and HELLS, respectively could cause immunodeficiency, centromeric instability, and facial anomalies (ICF) syndrome types 3 and 4 [28]. And the ZBTB24-CDCA7 axis could facilitate DNA methylation by regulating HELLS enrichment at centromeric satellite repeats [29]. As to the minichromosome maintenance (MCM) proteins family, they were mainly involved in the initiation and elongation of DNA replication [30] and the formation of the pre-replicative complex (preRC), the replication fork and the initial steps of DNA synthesis [31]. MCM2, MCM4, and MCM5 served as essential components of a hexameric, ring-shaped complex that acted as one of the pre-replication factors and were related to the chromatin and the proteins of the origin recognition complex at the M-G1 transition [30]. The important roles of these genes in the PPI network of CDCA7 all indicated the possible essential functions of CDCA7.

We also discovered the association of CDCA7 with immunity. CDCA7 was in close connection with the immune infiltration including B cell infiltration, CD4^+^ T cell infiltration, CD8^+^ T cell infiltration, neutrophil infiltration, macrophage infiltration, and dendritic cell infiltration. Moreover, CDCA7 was significantly correlated with tumor-related immunosuppressive molecules including PDCD1, CD274, CTLA4, BTLA and LAG3. PDCD1 (programmed cell death 1), CTLA-4 (cytotoxic T-lymphocyte-associated antigen 4) and BTLA (B and T lymphocyte attenuator) are members of immunoglobulin-related receptors family associated with various aspects of T cell immune regulation [32]. PDCD1 (PD-1, CD279) and CD274 (PD-L1) axis has been discovered as a worthy therapeutic target for its important role not only in physiological immune homoeostasis, but also in the way through which cancer cells evade the immune system [33]. Overexpression of PD-1 and PD-L1 in tumors is closely correlated with poor disease outcome in some human cancers [34]. The researches of PD-1 or PD-L1 inhibitors have greatly promoted the development of the treatment of cancer [35]. As to CTLA4, it is a transmembrane receptor with inhibitory function expressed by T lymphocytes. CTLA4 could inhibit costimulation through competing for CD28 ligands [36]. In addition to CTLA4 and PD1, BTLA is also an immune checkpoint related to suppress immune responses [37], containing two immunoreceptor tyrosine-based inhibitory motifs in its cytoplasmic region. BTLA could modulate T cell responses and attenuate B cell function to play its inhibitory roles in multiple diseases [38, 39]. Lymphocyte Activation Gene-3 (LAG3, CD223) served as another potential cancer immunotherapeutic target. It could inhibit T cells function and mediate a state of exhaustion in combination with PD1 [40]. The association of CDCA7 with these tumor-related immunosuppressive molecules suggested its important potential functions in human immunity.

Before fully understanding this article, several limitations should not be ignored. Firstly, clinical information was limited, due to the retrospective data from TCGA. Secondly, the sample sizes of normal renal tissue samples were relatively small (N = 72) in TCGA and this might lead to some biased in our conclusions. Thirdly, we currently could not tell the possible mechanism of CDCA7. Is it tumor intrinsic (related to tumor proliferation, migration, invasion?) or extrinsic (related to immune suppression?) or both? Future studies with larger sample sizes and sufficient clinical information were required to correct our results.

To conclude, our study elucidated that CDCA7 could act as a favorable prognostic factor for ccRCC. Moreover, apoptosis pathway, cell cycle pathway, JAK-STAT pathway, NOD like receptor pathway, P53 pathway, T cell receptor pathway and toll like receptor pathway might be the primary pathways regulated by CDCA7. Furthermore, CDCA7 could serve as an independent prognostic factor for ccRCC and it was closely related to MSI, TMB, and immunity. Further advanced researches in vivo and in vitro were required to verify our findings.

## Supplementary Information

**Additional file 1: Table S1**: Clinical information of the 16 ccRCC patients.

**Additional file 2: Figure S1**: The expressions of the significantly associated immune checkpoint molecules with CDCA7 in the TCGA dataset.

## Data Availability

The RNA-sequencing data and corresponding clinical information were downloaded from the Cancer Genome Atlas (TCGA) database (https://portal.gdc.cancer.gov/).
