# Supplementary material for: High expression of CDCA7 predicts poor prognosis for clear cell renal cell carcinoma and explores its associations with immunity
Source: Cancer Cell Int. 2021 Mar 1;21:140. doi: 10.1186/s12935-021-01834-x (PMC7923626; doi:10.1186/s12935-021-01834-x)
Supplement: Supplementary file 1 — Additional file 1: Table S1: Clinical information of the 16 ccRCC patients. [file 12935_2021_1834_MOESM1_ESM.docx]

**Table S1**: Clinical information of the 16 ccRCC patients;

| **ID** | **Gender** | **Age** | **Tumour Size (cm)** | **T** | **N** | **M** | **Stage** | **Grade** |
| --- | --- | --- | --- | --- | --- | --- | --- | --- |
| **ccRCC01** | Male | 51 | 4.0*2.0*3.5 | T1a | 0 | 0 | I | 1-2 |
| **ccRCC02** | Male | 45 | 3.5*2.8*2.3 | T1a | 0 | 0 | I | 1-2 |
| **ccRCC03** | Male | 36 | 2.5*2.0*2.0 | T1a | 0 | 0 | I | 2 |
| **ccRCC04** | Male | 75 | 4.5*3.5*3.5 | T1b | 0 | 0 | I | 2-3 |
| **ccRCC05** | Male | 56 | 10.0*9.0*9.0 | T2a | 0 | 0 | II | 1-2 |
| **ccRCC06** | Male | 74 | 5.0*4.8*2.8 | T1b | 0 | 0 | I | 1-2 |
| **ccRCC07** | Male | 54 | 6.0*4.5*4.0 | T1b | 0 | 0 | I | 2-3 |
| **ccRCC08** | Male | 26 | 2.5*2.0*2.0 | T1a | 0 | 0 | I | 1-2 |
| **ccRCC09** | Female | 59 | 9.0*7.0*5.0 | T4 | 1 | 1 | IV | 2 |
| **ccRCC10** | Male | 74 | 7.0*6.0*5.5 | T3b | 0 | 0 | III | 3-4 |
| **ccRCC11** | Male | 63 | 5.0*4.0*3.0 | T1b | 0 | 0 | I | 2-3 |
| **ccRCC12** | Male | 65 | 4.0*3.5*3.5 | T1a | 0 | 0 | I | 1-2 |
| **ccRCC13** | Female | 63 | 5.0*4.3*4.5 | T1b | 0 | 0 | I | 1 |
| **ccRCC14** | Male | 54 | 6.0*6.0*4.5 | T1b | 0 | 0 | I | 1-2 |
| **ccRCC15** | Male | 78 | 3.0*3.0*2.0 | T1a | 0 | 0 | I | 2 |
| **ccRCC16** | Male | 59 | 3.0*2.0*1.0 | T1a | 0 | 0 | I | 2 |
