# Supplementary figures and images for: High expression of CDCA7 predicts poor prognosis for clear cell renal cell carcinoma and explores its associations with immunity
Source: Cancer Cell Int. 2021 Mar 1;21:140. doi: 10.1186/s12935-021-01834-x (PMC7923626; doi:10.1186/s12935-021-01834-x)

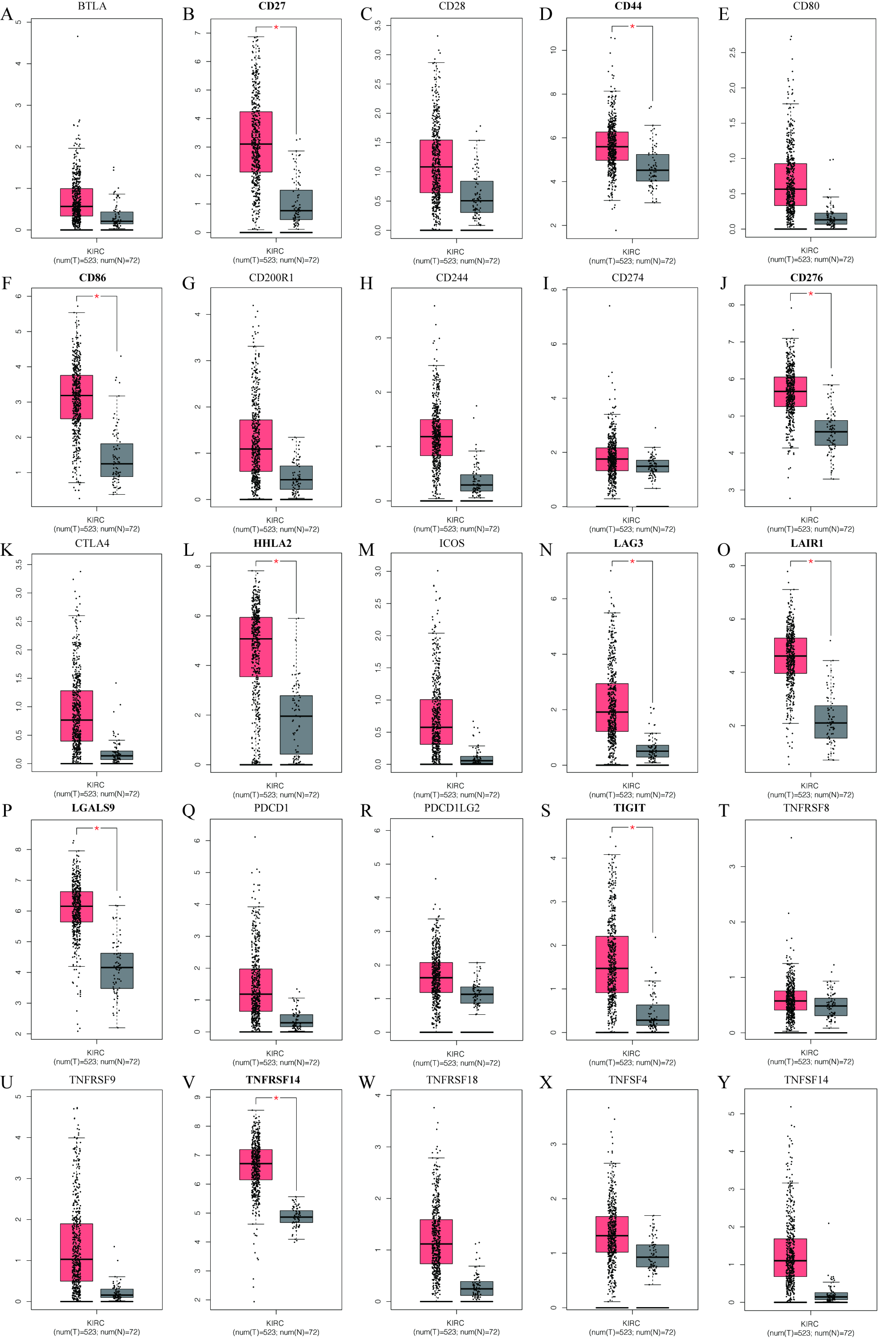

Supplement: Supplementary file 2 — Additional file 2: Figure S1: The expressions of the significantly associated immune checkpoint molecules with CDCA7 in the TCGA dataset. [file 12935_2021_1834_MOESM2_ESM.tif]
